# Successful Surgical Repair of Adult Anomalous Origin of the Left Main Coronary Artery from the Pulmonary Artery Complicated by a Mass in the Left Atrial Appendage: A Case Report

**DOI:** 10.70352/scrj.cr.24-0066

**Published:** 2025-03-21

**Authors:** Takashi Harada, Hironobu Morimoto, Yuki Echie, Daisuke Futagami, Keijiro Katayama, Shogo Mukai, Takaya Ozawa

**Affiliations:** 1 Department of Cardiovascular Surgery, Fukuyama Cardiovascular Hospital, Fukuyama, Hiroshima, Japan; 2 Department of Cardiology, Fukuyama Cardiovascular hospital, Fukuyama, Hirosima, Japan

**Keywords:** ALCAPA, Bland–White–Garland syndrome, adult, mass in the left atrial appendage, hypothermic circulatory arrest, LITA-LAD bypass

## Abstract

**INTRODUCTION:**

An anomalous origin of the left coronary artery (LCA) from the pulmonary artery (PA) (ALCAPA) is a rare congenital abnormality associated with high rates of early infant mortality and sudden death in adults.

**CASE PRESENTATION:**

A 56-year-old woman with acute left lower extremity arterial occlusion was diagnosed with an adult ALCAPA with a mass in the left atrial appendage. Preoperative echocardiography revealed left ventricular hypokinesis posteroinferior to the lateral wall and moderate mitral valve regurgitation, with a mass in the left atrial appendage. Coronary angiography revealed ALCAPA and dilatation of both coronary arteries. Myocardial scintigraphy revealed infarction of the posteroinferior wall and severe ischemia of the lateral wall of the left ventricle. We occluded the LCA entry from the inside of the PA and performed a left internal thoracic artery-to-left anterior descending artery (LITA-LAD) bypass, mitral annulus plasty, and resection of the mass together with the left atrial appendage. Because of residual myocardial blood flow from the collateral vessels, we cooled the temperature to 28°C to induce cardiac arrest. Postoperative coronary angiography indicated a good LITA-LAD flow and improvement in left ventricular contractility. Myocardial scintigraphy revealed improvement in ischemia. Pathological examination revealed that the mass in the left atrial appendage was a thrombus. The patient’s postoperative course was uneventful. She was discharged on postoperative day 16 and was given oral warfarin as anticoagulation therapy. Six months later, the follow-up evaluation was uneventful, and the patient was free of any symptoms of heart failure.

**CONCLUSIONS:**

We encountered a very rare case of ALCAPA complicated by a left intra-atrial mass following acute lower extremity artery occlusion. We performed LITA-LAD bypass, mitral annulus plasty, and resection of the mass along with the left atrial appendage. Due to residual myocardial blood flow from the collateral circulation, we cooled the temperature to 28°C in preparation for inducing cardiac arrest. The postoperative course was uneventful.

## Abbreviations

ALCAPA
: anomalous origin of the left coronary artery from the pulmonary artery
LCA
: left coronary artery
LITA-LAD
: left internal thoracic artery-to-left anterior descending artery
RCA
: right coronary artery

## INTRODUCTION

An anomalous origin of the left coronary artery (LCA) from the pulmonary artery (PA) (ALCAPA), also known as Bland–White–Garland syndrome, is a rare congenital abnormality associated with high rates of early infant mortality and sudden death in adults.

The incidence of ALCAPA is estimated to be 1 per 300000 live births. In rare cases, the development of collateral vessels to the LCA is adequate. These cases may progress asymptomatically into adulthood; however, the prognosis is considered poor owing to the development of heart failure and sudden death.^1)^

We herein report an extremely rare case of ALCAPA diagnosed at the onset of acute lower-extremity arterial occlusion caused by a mass in the left atrial appendage, which was surgically treated with a left internal thoracic artery-to-left anterior descending artery (LITA-LAD) bypass, mitral annulus plasty, and mass excision with left atrial appendage resection.

## CASE PRESENTATION

A 56-year-old woman complained of sudden left lower extremity pain and was diagnosed with an acute lower extremity arterial occlusion in the emergency room. The patient was healthy, had no comorbidities or significant medical history, and was not taking any medications.

**Fig. 1:**
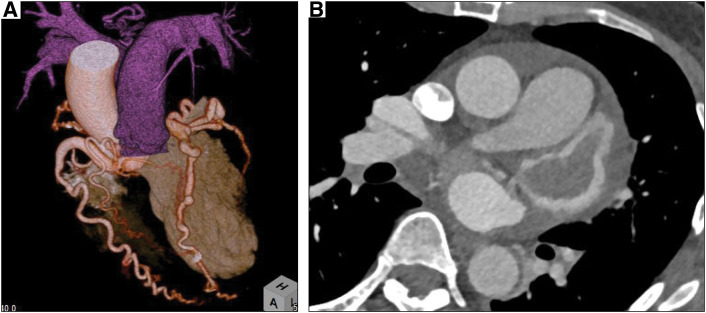
Computed tomographic reconstruction. The left coronary artery originated from the main pulmonary artery, with a mass in the left atrial appendage (**A** and **B**).

**Fig. 2:**
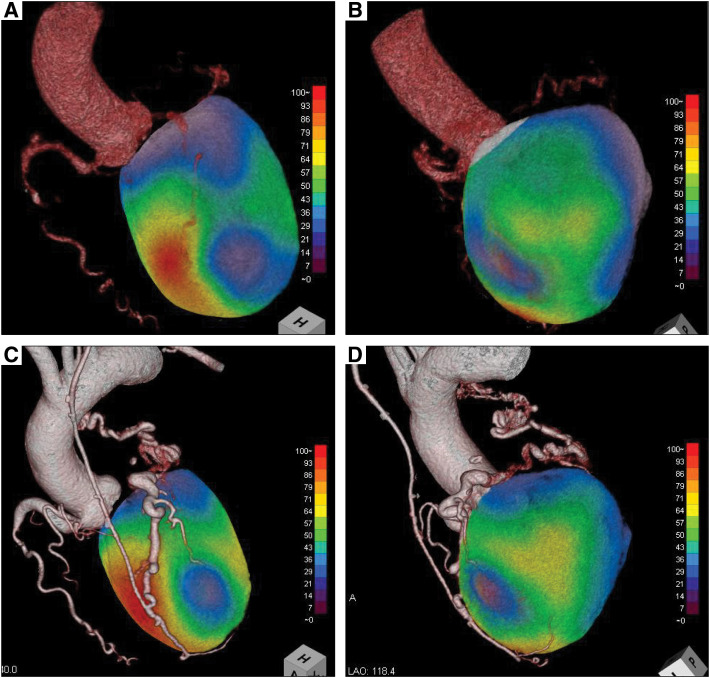
Technetium 99m sestamibi stress-rest myocardial perfusion scintigraphy. Exercise stress myocardial scintigraphy revealed infarction of the posterior inferior wall and severe ischemia of the lateral wall of the left ventricle (**A** and **B**). After the left internal thoracic artery-to-left anterior descending artery bypass, the myocardial ischemia from the lateral wall to the posterior wall of the left ventricle improved (**C** and **D**).

Thrombectomy was performed; however, preoperative echocardiography revealed a mass in the left atrium, left ventricular dysfunction posteroinferior to the lateral wall, and moderate mitral regurgitation. Multiple coronary computed tomography scans showed the origin of the LCA ostium in the PA and coronary artery dilatation (**Fig. 1**). The LCA was collateralized by the descending aorta and bronchial artery. Coronary angiography revealed that the enlarged right coronary artery (RCA) perfused the LCA, leading to the PA via the collateral vessels. Myocardial scintigraphy revealed infarction of the posteroinferior wall and severe ischemia of the lateral wall of the left ventricle (**Fig. 2**). Therefore, the patient was diagnosed with ALCAPA, complicated by moderate mitral regurgitation and a left atrial appendage mass.

We performed ALCAPA repair, mitral valve repair, and resection of the mass and left atrial appendage. Following median sternotomy, cardiopulmonary bypass was initiated with aortic and bicaval venous cannulations. Considering the difficulty of inducing cardiac arrest owing to the presence of collateral circulation from the systemic circulation to the coronary arteries, we cooled the temperature to 28°C to induce circulatory arrest for a brief period (within 5 minutes). After aortic cross-clamping, antegrade cold cardioplegia was administered to the aortic root, and the ostium of the LCA was temporarily occluded after opening the PA; cardiac arrest was achieved. Selective cardioplegia was administered to the ostium of the LCA, which arises from the left posterior sinus of the PA. We occluded the ostium of the LCA with mattress sutures using autologous pericardial pledgets, and antegrade cold cardioplegia was administered for subsequent myocardial protection. Myocardial protection of the LCA region in this adult ALCAPA case was deemed sufficient through RCA cardioplegia alone following ligation of the LCA orifice, given the well-developed collateral circulation from the RCA to the LCA.

**Fig. 3:**
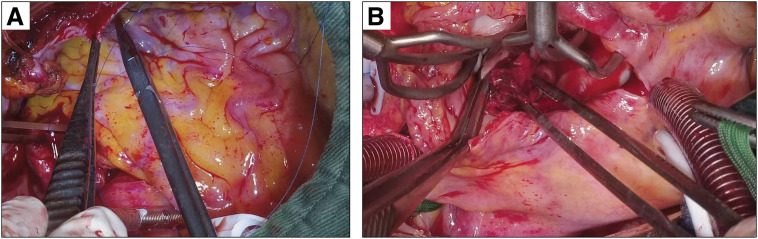
An intraoperative image shows multiple large collateral vessels arising from the right coronary artery due to the anomalous origin of the left coronary artery from the pulmonary artery. (**A**). Intraoperative findings of tumor in the left atrium (**B**). Pathological examination of the mass in the left atrial appendage revealed a thrombus.

We observed the left atrium using a transseptal approach and confirmed the mass on the left atrial appendage (**Fig. 3**). The mass was excised, and the left atrial appendage was resected using a linear cutter to prevent thrombus formation. The mitral annulus was dilated, and the posterior mitral leaflet was shortened and tethered to the left ventricle. We performed mitral annuloplasty using a 30-mm Physio II ring (Edwards Lifesciences, Irvine, CA, USA). Finally, we performed a LITA-LAD bypass; the flow of the bypass grafts was good, and transit-time flowmetry revealed a flow of 49 mL/min and a pulsatility index of 3.5.

The mass was initially suspected to be a neoplastic lesion before surgery; however, pathological examination revealed a thrombus. The patient did not have preexisting atrial fibrillation or episodes of atrial fibrillation during hospitalization.

The presence of moderate mitral regurgitation and a left ventricular ejection fraction (41%) due to ALCAPA likely contributed to blood stasis, which may have facilitated the formation of a thrombus within the left atrial appendage.

The postoperative course was uneventful. Transthoracic echocardiography and myocardial scintigraphy before discharge showed improved inferoposterior-to-lateral cardiac contractions and ischemia. Coronary angiography revealed that the LCA was contrasted not with the RCA but with the LITA without flow competition, and closure of the LCA ostium of the PA was confirmed. The patient was discharged on postoperative day 16 and was given oral warfarin as anticoagulation therapy. Six months later, the follow-up evaluation was uneventful, and the patient was free of any symptoms of heart failure.

## DISCUSSION

ALCAPA is an uncommon congenital coronary artery anomaly that typically presents in early infancy. Patients with ALCAPA are usually symptomatic, and ALCAPA in a previously asymptomatic patient diagnosed with acute lower extremity arterial occlusion with a mass in the left atrium is rare. The survival of patients with ALCAPA into adulthood has prompted the postulation of extensive collateral flow from the RCA to the LCA as a prerequisite. Blood flow into the low-pressure pulmonary circulation induces a left-to-right shunt-and-steal phenomenon, resulting in chronic ischemia, congestive heart failure, and sudden death;^1)^ hence, surgical correction of ALCAPA is recommended in all cases.^2)^

In ALCAPA, reimplantation of the LCA into the appropriate aortic sinus, with or without the use of an interpositional graft, is regarded as the optimal surgical strategy.^3–5)^ Coronary artery bypass graft with ALCAPA closure should be reserved only for patients in whom coronary transfer is not feasible. Establishing a two-coronary system is important.^6)^

In this case, we chose LCA ligation and LITA-LAD bypass because the distance from the LCA orifice to the ascending aorta was approximately 20 mm, which is relatively distant. The LCA ostium was enlarged to 8 mm, as the tissue flexibility in adult cases was poorer compared to pediatric cases, and the coronary artery appeared to be vulnerable.

While it might have been possible to consider intraoperative findings before finalizing the surgical plan, the procedure was predetermined due to the complexity of the operation, which included low cardiac function (ejection fraction 41%), LITA-LAD bypass, mitral annulus plasty, left atrial tumor resection, and left atrial appendage excision. Additionally, achieving a bloodless surgical field was anticipated to be challenging, and minimizing the aortic cross-clamp time was a critical consideration.

ALCAPA maintains coronary blood flow from the bronchial artery or other body circulations, even in the presence of an ascending aortic clamp.^7)^ As the visual field during intracardiac manipulation was expected to be poor and the time available for cardiac arrest was expected to be short, the temperature was cooled to 28°C to facilitate circulatory arrest. During the intracardiac procedure, there was a higher-than-usual backflow of blood, but the visual field remained accessible, and myocardial protection was required approximately every 15–20 minutes. Retrograde myocardial protection was considered an option in case antegrade myocardial protection failed to sustain cardiac arrest; however, it was not employed as the cardioplegia remained effective for approximately 15–20 minutes. The postoperative creatine kinase level was 467 IU/L at 10 hours, indicating that myocardial protection was adequate. The aortic cross-clamp time during surgery was 160 minutes, and the cardiopulmonary bypass time was 226 minutes. The LITA graft flow measured at the time of surgery was 49 mL/min, which was deemed adequate.

While more flow could have been achieved with an saphenous vein graft-left anterior descending artery bypass or a bypass to the left main trunk using an artificial graft, we opted for a LITA graft due to its high patency rate and considering the patient’s age of 56 years.

Although transthoracic echocardiography and myocardial scintigraphy before discharge showed improved inferoposterior-to-lateral cardiac contractions and ischemia (**Fig. 3**), future complications such as graft failure are possible; thus, rigorous follow-up is necessary.

## CONCLUSIONS

ALCAPA is often diagnosed by symptoms of heart failure such as shortness of breath, chest pain and syncope, and we experienced a very rare case of ALCAPA complicated by a left intra-atrial mass following acute lower extremity artery occlusion. Although a LITA-LAD bypass was performed and blood flow to the left ventricular myocardium sufficiently improved, long-term follow-up is required. There are limited reports on the surgical treatment of ALCAPA in adults, and the etiology of the mass in the atrial appendage remains unclear.

## ACKNOWLEDGMENTS

We would like to thank Editage (www.editage.jp) for English language editing.

## DECLARATIONS

### Funding

The study received no external funding.

### Authors’ contributions

TH: writing papers, coordinating with journals, and taking responsibility.

HM, YE, DF, KK, SM, and TO: clinical practice and data organization.

Final approval of the article: all authors.

Description of all aspects of the work: all authors.

All authors agree to take responsibility for all aspects of the study.

### Availability of data and materials

The datasets used and/or analyzed during the current study are available from the corresponding author upon reasonable request.

### Ethics approval and consent to participate

The patient provided written informed consent, and the study design was approved by the ethics review board of Fukuyama Cardiovascular Hospital Ethics Committee (approval no: 111).

### Consent for publication

We have obtained written informed consent from the patient for the publication of this case report and images.

### Competing interests

The authors declare that they have no competing interests.
